# Ipatasertib, a novel Akt inhibitor, induces transcription factor FoxO3a and NF-κB directly regulates PUMA-dependent apoptosis

**DOI:** 10.1038/s41419-018-0943-9

**Published:** 2018-09-05

**Authors:** Li Sun, Yuan Huang, Yeying Liu, Yujie Zhao, Xiaoxiao He, Lingling Zhang, Feng Wang, Yingjie Zhang

**Affiliations:** 1 College of Biology, Hunan University, Changsha, 410082 China; 2 Department of Gastroenterology, The Tenth People’s Hospital of Shanghai, Tongji University, Shanghai, 200072 China; 3 Department of Laboratory Medicine, Xiangya School of Medicine, Central South University, Changsha, 410013 China; 4 Shenzhen Institute, Hunan University, Shenzhen, China; 5 Department of Out-patient, Affiliated Hospital of Hebei University of Engineering, Handan, 056002 China

## Abstract

Colon cancer is one of the three common malignant tumors, with a lower survival rate. Ipatasertib, a novel highly selective ATP-competitive pan-Akt inhibitor, shows a strong antitumor effect in a variety of carcinoma, including colon cancer. However, there is a lack of knowledge about the precise underlying mechanism of clinical therapy for colon cancer. We conducted this study to determine that ipatasertib prevented colon cancer growth through PUMA-dependent apoptosis. Ipatasertib led to p53-independent PUMA activation by inhibiting Akt, thereby activating both FoxO3a and NF-κB synchronously that will directly bind to PUMA promoter, up-regulating PUMA transcription and Bax-mediated intrinsic mitochondrial apoptosis. Remarkably, Akt/FoxO3a/PUMA is the major pathway while Akt/NF-κB/PUMA is the secondary pathway of PUMA activation induced by ipatasertib in colon cancer. Knocking out PUMA eliminated ipatasertib-induced apoptosis both in vitro and in vivo (xenografts). Furthermore, PUMA is also indispensable in combinational therapies of ipatasertib with some conventional or novel drugs. Collectively, our study demonstrated that PUMA induction by FoxO3a and NF-κB is a critical step to suppress the growth of colon cancer under the therapy with ipatasertib, which provides some theoretical basis for clinical assessment.

## Introduction

Colon cancer is a common occurrence in a malignant tumor of the digestive tract with the high mortality rate, but low survival rate^1^. Many significant therapeutics over the past decade have been used for colon cancer therapy^2,3^. However, chemotherapeutic agents gradually show their drawbacks due to lack of specificity^4^. Drugs with the specific target are now being developed for clinical application to treat human colon cancer. Ipatasertib, also known as GDC-0068, a novel highly selective ATP-competitive pan-Akt inhibitor that inhibits all three isoforms of Akt, was identified by structure-guided drug design^5^. Ipatasertib was evaluated in human cancer cell lines and xenograft models with various genetic backgrounds, either as a single agent or in combination with chemotherapeutic agents, such as docetaxel and carboplatin^6^. As an ATP-competitive Akt inhibitor, ipatasertib is a strong and safe target for Akt, which has been proved in a first-in-human phase I study^7^. However, few studies have been done on the mechanism of tumor suppression induced by ipatasertib.

PMUA, (p53 Upregulated modulator of apoptosis) (PUMA), a Bcl-2 homology 3 (BH3)-only Bcl-2 family member, plays an essential role in p53-dependent and -independent cell apoptosis induced by a variety of stimuli, including inhibited de-regulated oncogene expression, genotoxic stress, growth factor/cytokine withdrawal and infection, toxins, altered redox status^8–12^. PUMA maintains a relatively low level in normal human cells and tissue, but it will be up-regulated by activated p53 in most of the human tumors. Once activated by apoptotic stimuli, PUMA directly induces Bax/Bak mitochondrial membrane translocation and activates these proapoptotic signals, which cause mitochondrial outer membrane permeabilization (MOMP), caspase cascade, and apoptosis^9,12–15^. In the study of acute lymphocytic leukemia (ALL), PUMA induced by some dual mTOR inhibitors is a major determinant in killing ALL cells, via mTORC1/4EBP1/MYC/PUMA pathway^16^. Research has identified that PUMA plays a crucial role in the miRNA-induced drug resistance of colorectal carcinoma (CRC)^17^. Mitochondrial apoptosis initiated by various stimuli will abrogate after the knockdown of PUMA in the human malignant tumor, including colon cancer^18^. In contrary, over-expressing PUMA enormously enhances apoptosis and inhibits tumor growth^19^. Our previous study has proved that PUMA was transcriptionally activated by FoxO3a following Akt inhibition^20^ or by NF-κB following PI3K inhibitor^21^ in colon cancer and played a crucial role in drug-induced apoptosis.

Akt, a serine/threonine-specific protein kinase that plays a key role in a variety of cellular processes such as glucose metabolism, apoptosis, cell proliferation, transcription, and cell migration, is continuously activated in various human tumors^22^. As noted above, dysregulation of the PI3K/Akt pathway cooperates with many human malignancies. For example, mutation of Akt-activated is contributed to increase the risk of colon cancer^23^; Akt signaling activated by YEP promotes mitotic arrest, polyploidy, and hepatocellular carcinoma^16^; hyperactivation of Akt depletes hematopoietic stem cells and induces leukemia in mice^24^. Akt, as the major regulatory factor in PI3K/Akt signaling pathway, once activated, phosphorylates its myriad substrates (e.g., FoxO3a, NF-κB, mTOR, VEGF) via its kinase activity. Forkhead Box O3a (FoxO3a), a recently discovered FOXO family protein, contains a special DNA binding domain. FoxO3a is the common substrate molecule in PI3K/Akt signaling pathway. FoxO3a phosphorylated by Akt inhibits its transcriptional function that deregulates pro-apoptotic genes and promotes cell survival. The blockage of PI3K/Akt signaling pathway effectively activates nuclear translocation of FOXO transcription factors that target the PUMA promoter to transactivate PUMA, triggering apoptosis and inhibiting tumors progression^13,25–27^. Nuclear factor κB (NF-κB), nuclear factor kappa-light-chain-enhancer of activated B cells, is composed of p50, p52, p65 (or RelA), c-Rel, or RelB subunits, which forms functional protein complex after dimerization^28^. Akt directly or indirectly regulates the activity of IKK (the IκB kinase), leading to the nuclear translocation and activation of NF-κB^29^. It has been demonstrated that one downstream effect of Akt activation is NF-κB-dependent transcription and numerous indications of interaction between the Akt and NF-κB pathways in human malignant tumor including colon cancer^30,31^. NF-κB, as a transcription factor that regulates intracellular factor production and cell survival, directly regulates PUMA and induces PUMA-dependent apoptosis in vitro and in vivo^20,32^. PUMA is induced by regorafenib through the NF-κB pathway, which plays a pivotal role in therapeutic response to regorafenib in colorectal cancer cells^33^.

Previous reports from our laboratory and others have revealed that PUMA activates Bax and triggers the intrinsic mitochondrial apoptosis pathway after inhibition of PI3K/Akt by anti-tumor drugs or inhibitors^20,34^. In this present study, ipatasertib was utilized to suppress colon cancer growth and the molecular mechanism was clarified. Our results show that ipatasertib induced colon cancer cell apoptosis by activating PUMA, which was dependent on FoxO3a and NF-κB, but not p53. PUMA was required for ipatasertib therapy only and combinational therapies of ipatasertib and other drugs in colon cancer. These results make PUMA as a potential indicator of ipatasertib therapeutic efficacy, as well as other drugs.

## Results

### Ipatasertib suppressed colon cancer cell proliferation by p53 irrespectively activating PUMA

**Fig. 1:**
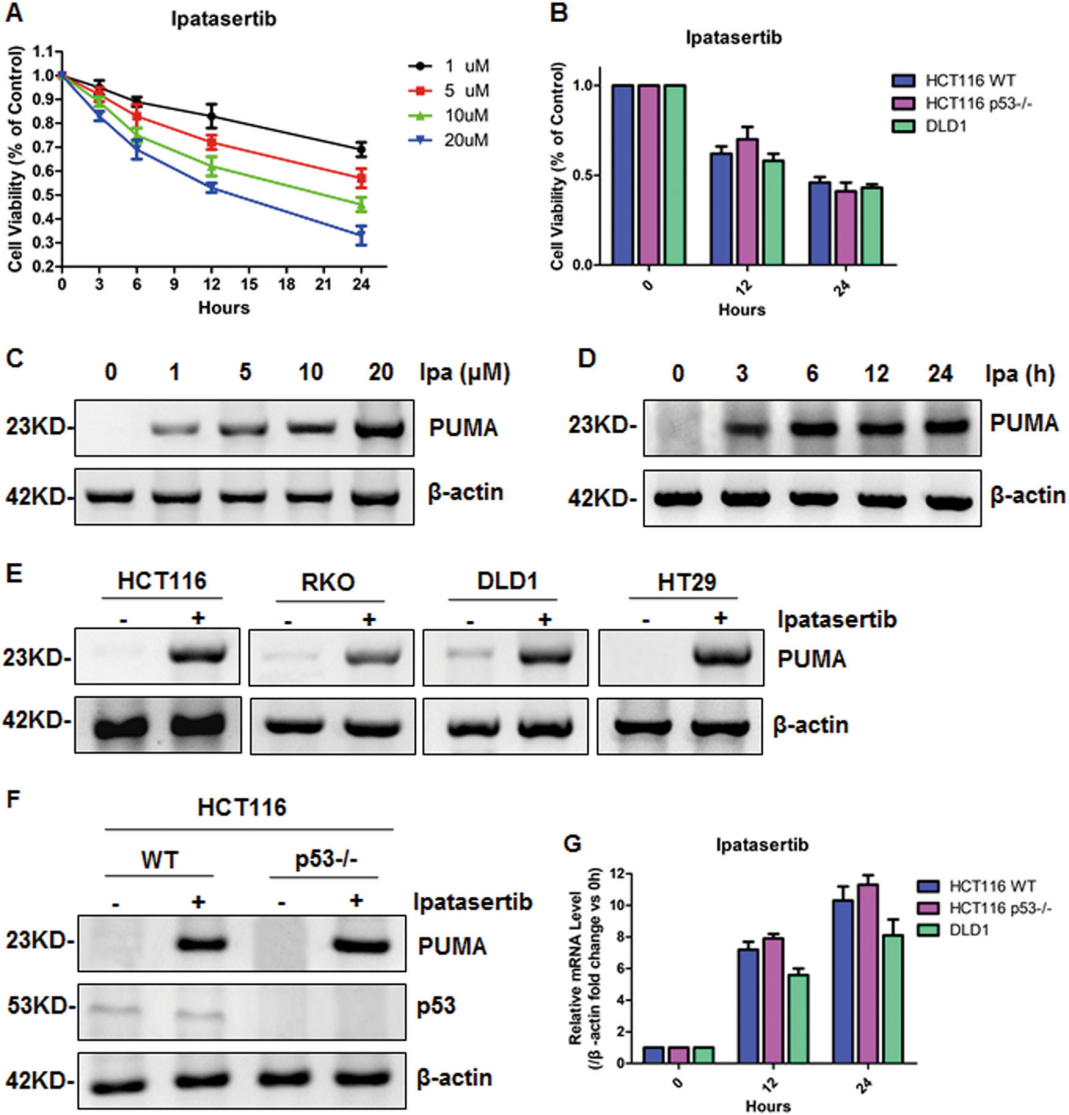
Ipatasertib promoted cell apoptosis and PUMA induction in colon cancer cells. **a** Cell viability of HCT116 was analyzed using Cell Counting Kit-8 at 0, 3, 6, 12, and 24 h after 1, 5, 10, or 20 μM ipatasertib treatment. **b** Cell viability was analyzed using Cell Counting Kit-8 at 0, 12, and 24 h after 10 μM ipatasertib treatment in HCT116 WT, p53^−/−^, and DLD1. Data represent the mean ± SEM of four independent experiments. **c–g** Ipatasertib increased p53-independent PUMA expression in various colon cell lines. **c**, **d**, **e** Western blotting analysis of PUMA expression in HCT116 treated with 1–20 μM for 24 h (**c**) and with 10 μM ipatasertib for indicated hours (**d**) or in various colon cancer cell lines treated with 10 μM ipatasertib for 24 h (**e**). **f** The expression of p53 or PUMA by western blotting analysis in HCT116 WT and p53^−/−^ treated with 10 μM ipatasertib for 24 h. **g** PUMA mRNA induction by ipatasertib was analyzed in WT, p53^−/−^HCT116, and DLD1 by real-time qPCR and normalized to the housekeeping gene β-actin. The values are the mean ± SEM (*n* = 3) from a representative experiment

To study how ipatasertib influences tumor progression, cell viability was detected by CCK-8 in HCT116 at indicated time points after 1–20 μmol/L ipatasertib treatment. As a result, cell viability decreased markedly with increasing dose or time (Fig. 1a), suggested that cell proliferation was inhibited by ipatasertib in a dose- and time-dependent manner. In order to explore whether p53 has a role in this process, HCT116 WT, p53^−/−^, and DLD1 (p53 mutant) cells were treated with 10 μmol/L ipatasertib for 12 and 24 h. As shown in Fig. 1b, all the three cell lines decreased obviously in viability, indicated p53 is dispensable. Next, the IC50 values of ipatasertib in HCT116 WT and p53^−/−^ cells were calculated by using CCK-8 (10.58 and 9.149 μmol/L, Figure S1A and S1B, Table S1A and S1B), which suggested that 10 μmol/L was an appropriate dose for inhibiting colon cancer cell proliferation and p53 played little function in this process. To study the effect of ipatasertib in normal colon cell line, NCM460 cells were treated with various doses and the results show no big difference (Figure S1C). In addition, another ATP-competitive Akt inhibitor afuresertib and allosteric Akt inhibitor perifosine were conducted to treat HCT116 cells, as shown in Figure S1D, both of them obviously inhibited cell proliferation.

As an important apoptotic promoter and tumor suppressor, PUMA was tested whether had a response to ipatasertib treatment. The results show that PUMA expression level was up-regulated gradually with increasing dose (Fig. 1c) or time post-ipatasertib treatment (Fig. 1d). This up-regulation was observed in both WT (HCT116, RKO), p53 mutant (DLD1, HT29) and p53^−/−^ (HCT116 p53^−/−^) colon cancer cells (Fig. 1e. f). Interestingly, p53 expression had no change at all even treated with ipatasertib (Fig. 1f), suggested it had no effect on PUMA induction during this process. The mRNA level of PUMA was also detected with different p53 statuses, and it was enhanced significantly in all the three cell lines (Fig. 1g). Taken together, all these data above indicated that ipatasertib treatment resulted in a p53-independent transcriptional activation of PUMA and inhibition of cell proliferation.

### FoxO3a and p65 were activated with PUMA up-regulation after Akt inhibition by ipatasertib

**Fig. 2:**
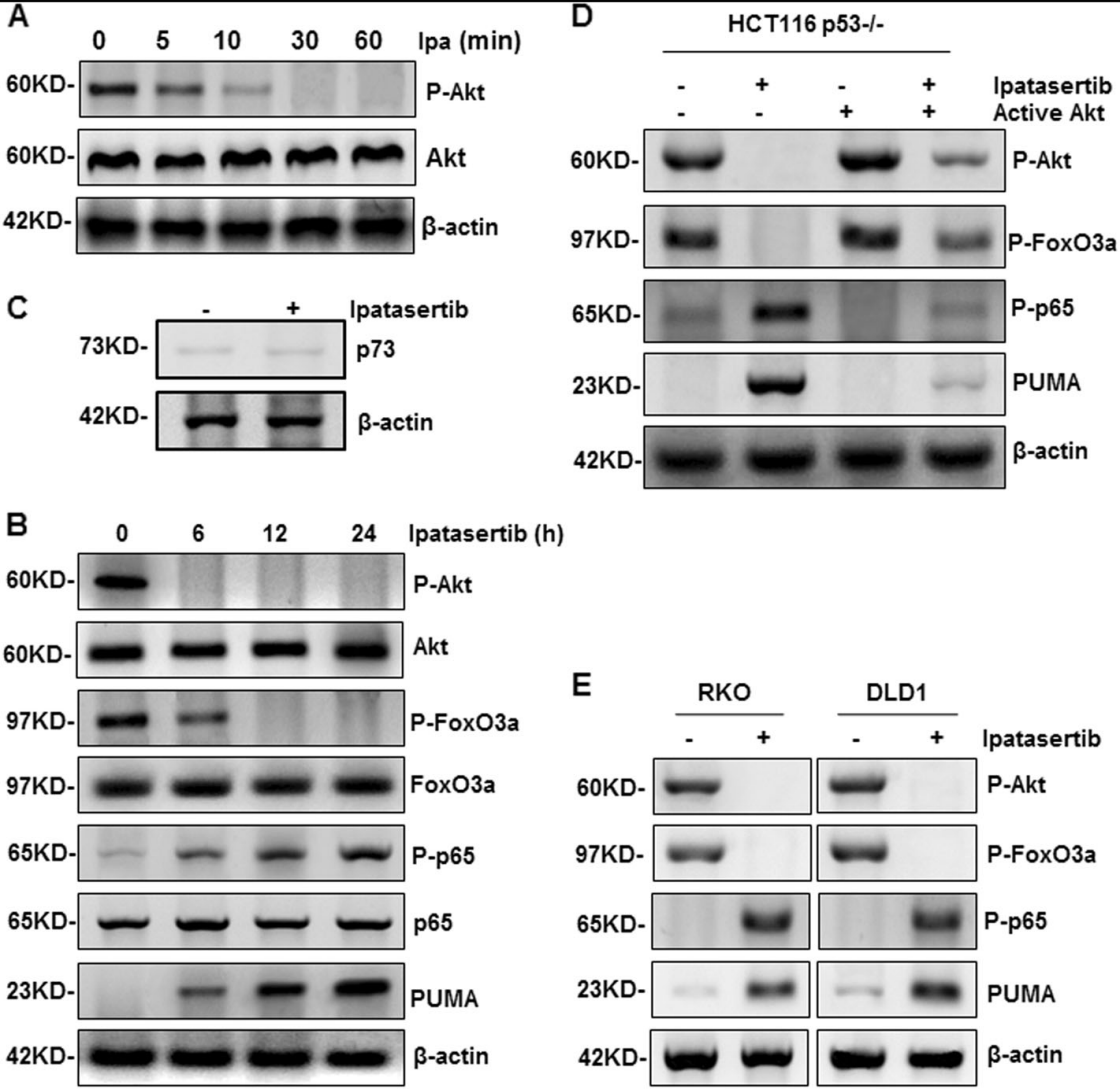
Ipatasertib activated FoxO3a and p65 by Akt inhibition. **a** Akt was inhibited after ipatasertib treatment in HCT116 with 10 μM ipatasertib treatment for indicated times. **b** The expressions of P-Akt (S473), P-FoxO3a (S253), P-p65(S536), and PUMA were detected after the treatment of 10 μM ipatasertib in HCT116 at 0, 6, 12, and 24 h after 10 μM ipatasertib treatment. **c** The expression of p73 was detected by western blotting in HCT116 with ipatasertib treatment. **d** HCT116 p53^−/−^ were transfected with either empty vector or a constitutively-active Akt expression constructs for 24 h, following 24 h of 10 μM ipatasertib treatment. The levels of P-Akt (S473), P-FoxO3a (S253), P-p65(S536), and PUMA were analyzed by western blotting. **e** Western blotting shows the expression of P-Akt (S473), P-FoxO3a (S253), P-p65(S536), and PUMA in RKO and DLD1 cells after 10 μM ipatasertib treatment or not

Ipatasertib, a novel Akt inhibitor, takes pharmacological effect through inhibiting Akt activation. As shown in Fig. 2a, the phosphorylation level of Akt obviously decreased in HCT116 WT cells at indicated time points and disappeared within 1 h after ipatasertib stimulation. Consistent with our previous results, PUMA expression was enhanced evidently (Fig. 2b). In parallel, the phosphorylation level of FoxO3a declined significantly while that of p65 increased gradually (Fig. 2b and Figure S2A), which indicated that both FoxO3a and p65 were activated synchronously after stimulation by ipatasertib. Similar results were obtained from HCT116 p53^−/−^ (Fig. 2d), RKO and DLD1 (p53 mutant) cells (Fig. 2e). As another direct transcriptional factor of PUMA, p73 was also detected. As a result, ipatasertib treatment did not change p73 expression level (Fig. 2c), indicating p73 was not activated and had no relation with PUMA up-regulation. Next, active Akt was over-expressed in HCT116 p53^−/−^ cells and markedly abolished ipatasertib-induced decrease of phosphorylated Akt and FoxO3a, as well as the increase of PUMA and phosphorylated p65 (Fig. 2d). In addition, ipatasertib treatment did not cause Akt inactivation, or the activation of FoxO3a, p65, and PUMA in NCM460 cells (Figure S2B). Afuresertib enhanced a few activities of FoxO3a, p65, and PUMA, but not as significant as that of ipatasertib, while perifosine had no effect on these events at all (Figure S2C). These results suggested that Akt inactivation by ipatasertib activates both FoxO3a and p65 simultaneously and up-regulates PUMA expression in colon cancer cells regardless of p53 status.

### FoxO3a and p65 transcriptionally activated PUMA after ipatasertib stimulation

**Fig. 3:**
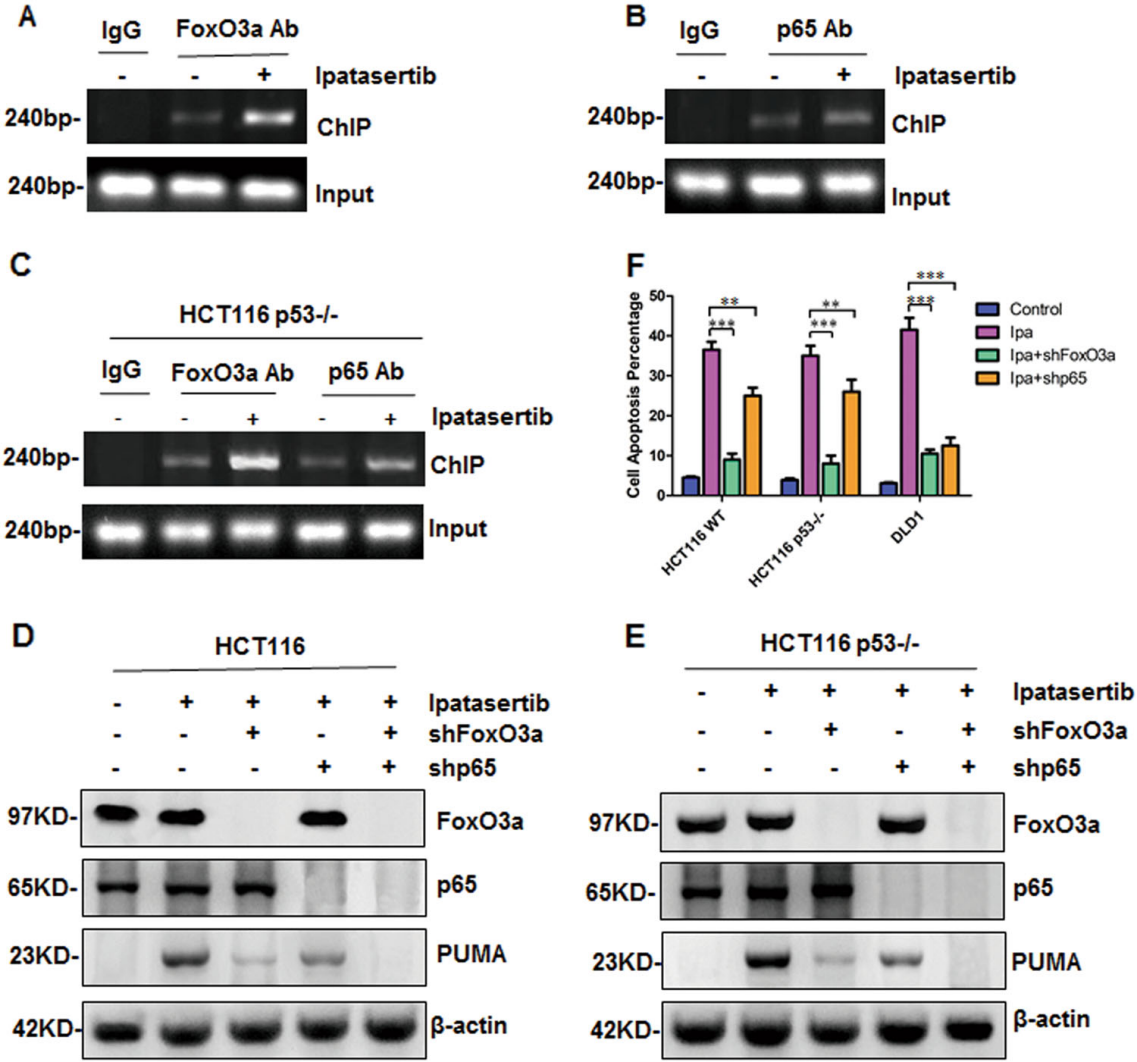
Both FoxO3a and p65 regulated PUMA induction by ipatasertib. **a**–**c** Chromatin immunoprecipitation (ChIP) was performed on HCT116 WT and p53^−/−^ after 12 h ipatasertib treatment. IgG was used as a control for the FoxO3a-specific antibody and the p65-specific antibody. **d**–**f** The effect of FoxO3a or p65 knockdown on colon cancer cells. **d**, **e** FoxO3a, p65, and PUMA were detected in HCT116 WT or p53^−/−^ following the treatment of 10 μM ipatasertib, with FoxO3a or p65 knockdown or not. Similar results were obtained from three independent experiments. **f** Cell apoptosis was detected by nuclear staining with Hoechst 33258 in HCT116 WT, p53^−/−^, and DLD1 after treated with ipatasertib for 24 h, with FoxO3a and p65 knockdown or not. Similar results were obtained from three independent experiments. Data represent the mean ± SEM of four independent experiments. **P < 0.01, ***P < 0.001 vs. FoxO3a and p65, not a knockdown

Previous data indicated that FoxO3a and p65 may serve as transcriptional factors to regulate PUMA expression. To demonstrate it, chromatin immunoprecipitation (ChIP) assay was performed in HCT116 cells and the results show both FoxO3a and p65 had an increasing binding with PUMA promoter (Fig. 3a, b), and FoxO3a shows stronger interaction with PUMA promoter in this process. Next, to exclude the influence of p53 and compare FoxO3a and p65 directly, same experiments were performed in HCT116 p53^−/−^ cells at the same time, and almost identical results were obtained (Fig. 3c), indicating that both FoxO3a and p65 can bind to PUMA promoter and regulate PUMA expression directly. To further confirm this result, FoxO3a or p65 was knocked down by using shRNAs in HCT116 WT and p53^−/−^ cells. As a result, either knocking down FoxO3a or p65 obviously eliminated ipatasertib-induced PUMA up-regulation in these two different cell lines (Fig. 3d, e). Meantime, knocking down FoxO3a shows more powerful influence on PUMA up-regulation than that of knocking down p65 (Fig. 3d, e). However, knocking down both FoxO3a and p65 simultaneously totally abolished ipatasertib-induced PUMA up-regulation, which shows a superposed effect (Fig. 3d, e). Furthermore, cell apoptosis was also detected in different conditions, and similar trends were obtained. Knocking down FoxO3a significantly eliminated ipatasertib-induced apoptosis in HCT116 WT, p53^−/−^, and DLD1 cells (Fig. 3f). For knocking down p65, it had an obvious effect in DLD1 cells, but not so significant in HCT116 WT and p53^−/−^ cells. Together, these results suggested that ipatasertib treatment inhibited Akt, then activated both FoxO3a and p65, subsequently binded to PUMA promoter and up-regulated its expression, finally promoted apoptosis, during which FoxO3a is dominant while p65 is the secondary regulator.

### Ipatasertib induced apoptosis through PUMA/Bax pathway

**Fig. 4:**
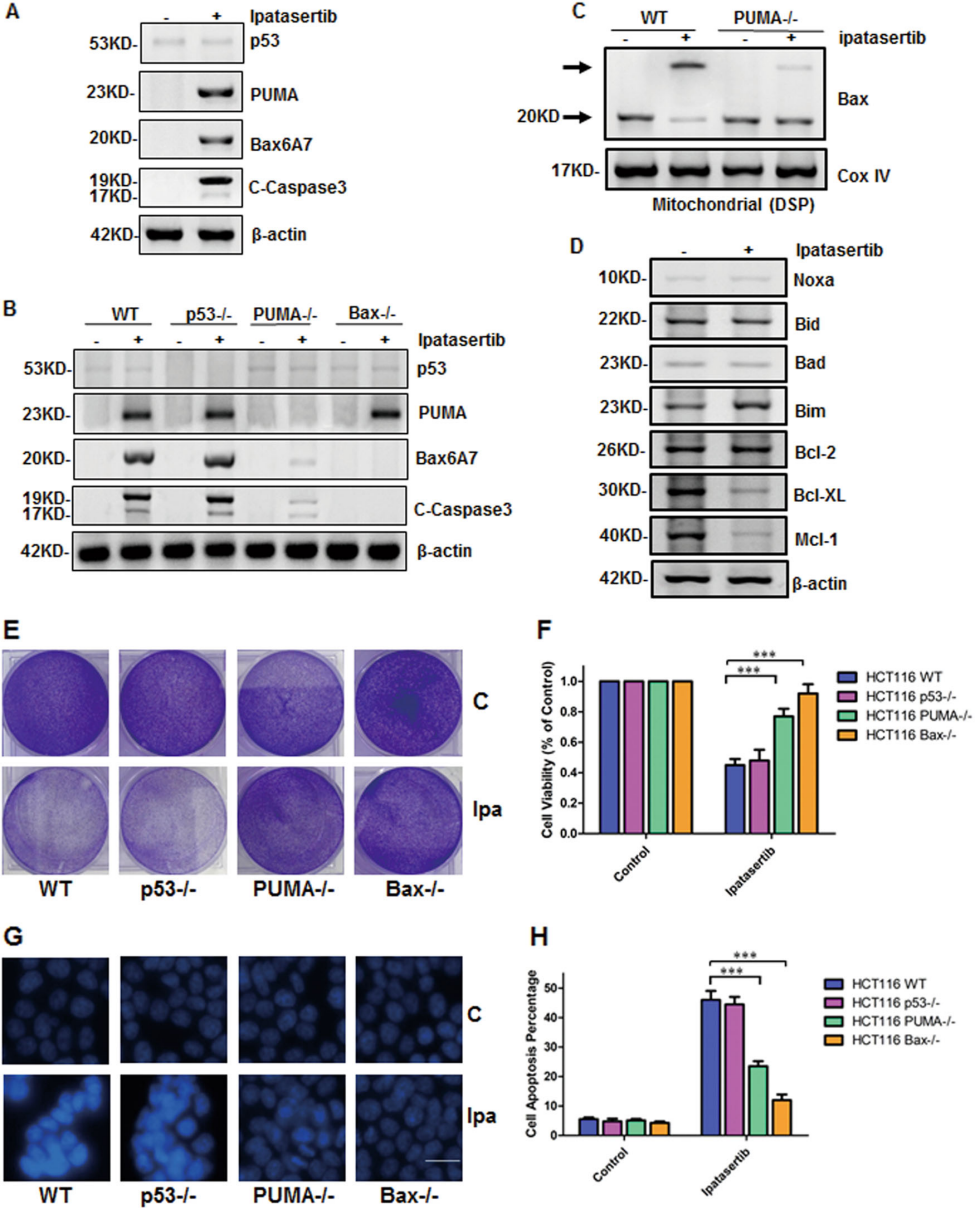
Ipatasertib induced PUMA/Bax dependent apoptosis. **a**, **b** Western blotting shows the expression of p53, PUMA, Bax6A7, and cleaved caspase3 in HCT116 (**a**) or in WT p53^−/−^, PUMA^−/−^, and Bax^−/−^ (**b**) with 10 μM ipatasertib for 24 h. **c** Bax multimerization in mitochondria fraction was analyzed by Western blotting after DSP cross-link. **d** Noxa, Bid, Bad, Bim, Bcl-2, Bcl-XL, Mcl-1 were detected in HCT-116 cells after ipatasertib for 24 h. **e** Colony formation was analyzed by crystal violet staining in HCT116 WT, p53^−/−^, PUMA^−/−^, and Bax^−/−^ after 24 h of ipatasertib treatment. **f** CCK-8 assay was performed to show colon cancer cells’ viability after p53, PUMA, or Bax knockdown in HCT116 with ipatasertib treatment for 24 h. **g** Hoechst 33258 morphological examination of apoptosis in HCT116 WT, p53^−/−^, PUMA^−/−^, or Bax^−/−^. Cells were treated with 10 μM ipatasertib and incubated for 24 h, then stained with Hoechst 33258. **h** Cell apoptosis was analyzed by fluorescence-activated cell sorting (FACS) analysis after HCT116 cells were treated with 10 µM ipatasertib for 24 h. The percentage of apoptotic cells was calculated from FACS analysis. Data represent the mean ± SD of three independent experiments. ***P < 0.001 vs. untreated control cells

Our previous studies have proved that PUMA directly and indirectly activates Bax to induce apoptosis^35^. In this study, to exemplify whether ipatasertib induces apoptosis via PUMA/Bax axis, PUMA, Bax6A7, and C-Caspase3 were firstly detected in HCT116 cells, and all of them increased whereas p53 had nearly no change (Fig. 4a).

However, in the HCT116 PUMA^−/−^ cells, Bax activation and cleavage of Caspase 3 were markedly abolished, which means PUMA is required (Fig. 4b). To further confirm PUMA-mediated Bax activation, Bax oligomerization in mitochondria was detected. Consistent with the results of Fig. 4a, b, Bax dimerization increased after ipatasertib treatment which was almost abrogated in PUMA^−/−^ cells (Fig. 4c). Furthermore, Bax deficiency totally eliminated Caspase 3 activation even in the presence of PUMA (Fig. 4b), which suggested that Bax is indispensable. Besides, other Bcl-2 family members were detected and are shown in Fig. 4d. The expression of Noxa, Bid, Bad, and Bcl-2 had no change in the presence of ipatasertib, Bim increased but not so markedly as that of PUMA, Bcl-XL, and Mcl-1 decreased evidently. LC3-II and P-MLKL were also detected to investigate whether autophagy or necroptosis happened with apoptosis at the same time, and negative results were obtained (Figure S3A).

To observe cell growth status in the different genetic background, colony formation assay was performed in HCT116 WT, p53^−/−^, PUMA^−/−^, and Bax^−/−^ cells. As a result, p53 deficiency did not affect ipatasertib-induced cell growth inhibition, which was significantly abolished in PUMA and Bax deficiency cells (Fig. 4e). Cell viability detection gave us the similar trends (Fig. 4f). Finally, the morphology of apoptosis was studied by using Hoechst 33258 staining. The results show that p53 deficiency had no influence on ipatasertib-induced apoptosis, which was obviously eliminated in HCT116 PUMA^−/−^ and Bax^−/−^ cells (Fig. 4g). In parallel, the results from the statistical analysis show an identical trend (Figure S3B). Flow cytometry experiments were also performed to further confirm the previous results and got a similar conclusion (Fig. 4h and Figure S3C). In short, these results revealed that PUMA/Bax axis is indispensable for ipatasertib-induced apoptosis, which is through the mitochondrial pathway.

### PUMA is required for combinational therapies in colon cancer cells

**Fig. 5:**
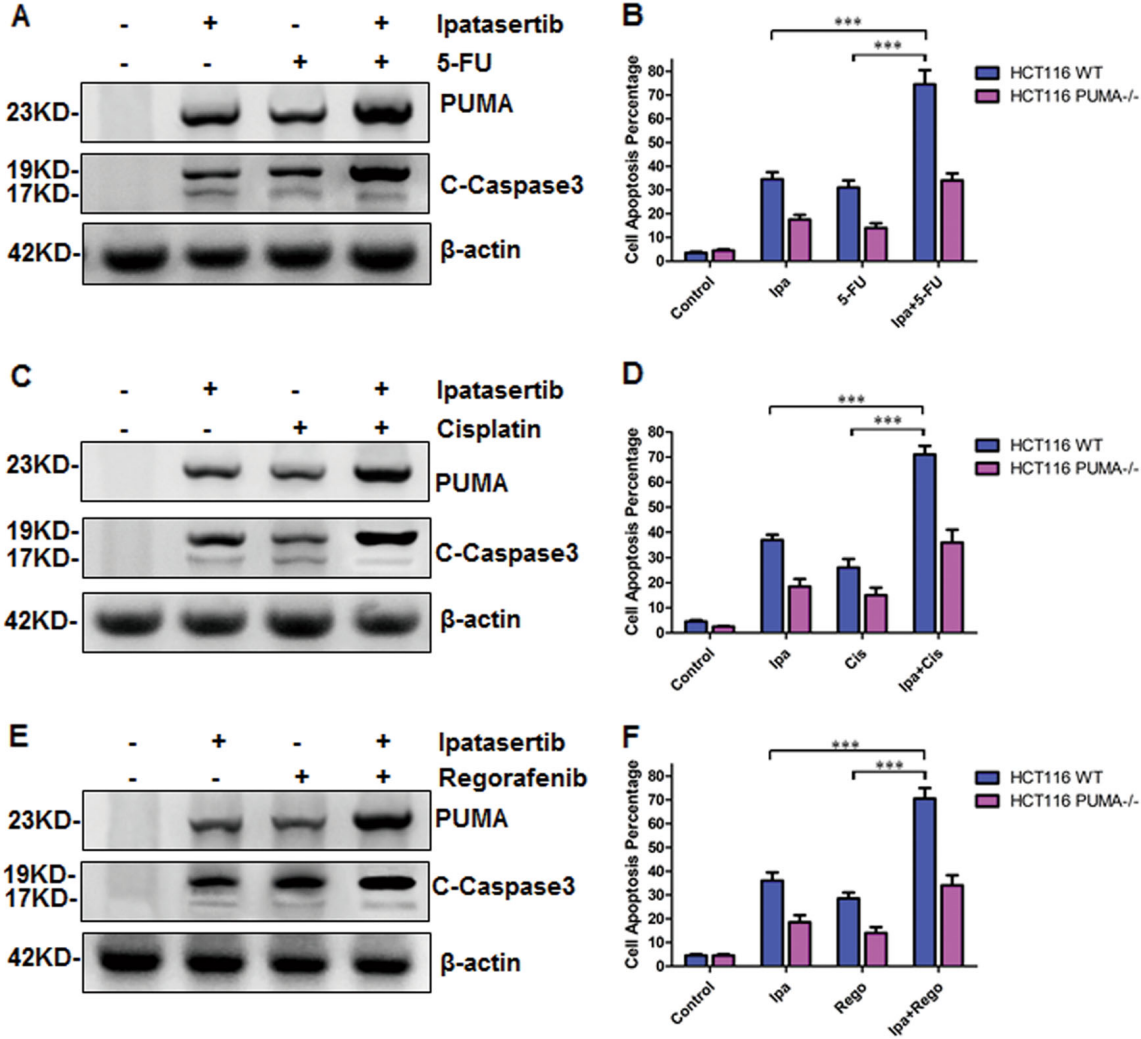
Ipatasertib increased the chemosensitization effects by inducing PUMA-dependent apoptosis. **a**, **c**, **e** Western blotting analysis of PUMA and C-Caspase3 expression after the treatment of 10 μM ipatasertib in combination with **a** 20 mg/ml 5-FU or **c** 40 μM Cisplatin or **e** 20 mM Regorafenib alone, or their combinations for 24 h in HCT116. **b**, **d**, **f** Apoptosis was analyzed by nuclear staining with Hoechst 33258 in HCT116 WT or PUMA^−/−^ treated with **b** ipatasertib and 5-FU alone or their combinations as (**a**), **d** Ipatasertib and Cisplatin alone or their combinations as (**c**), or **f** Ipatasertib and Regorafenib alone or their combinations as (**e**). Data represent the mean ± SEM of four independent experiments. ***P < 0.001 vs. alone

Akt activation has been associated with resistance to chemotherapeutic agents^36,37^. Ipatasertib combined with multiple chemotherapeutic agents have a synergistic effect in a variety of cancer cell lines^29,38^. PUMA-dependent apoptosis induced by ipatasertib have been approved in the preceding analysis. To unravel the therapeutics of ipatasertib, the combinational treatments of ipatasertib with other drugs that induce PUMA expression and apoptosis, such as 5-FU, Cisplatin, or Regrafenib were performed in HCT116 cells. The results show that the combinational treatments further up-regulated the expression of PUMA and C-Caspase3 (Fig. 5a, c, e). Statistical analysis shows that combinational therapies of ipatasertib with 5-FU, Cisplatin, or Regrafenib further enhanced cell apoptosis compared with that of ipatasertib treatment only, which was significantly blocked by PUMA deficiency (Fig. 5b, d, f). These data mean PUMA plays a crucial role in the combinational therapies for colon cancer cells.

### The antitumor activity of ipatasertib depends on PUMA in vivo

**Fig. 6:**
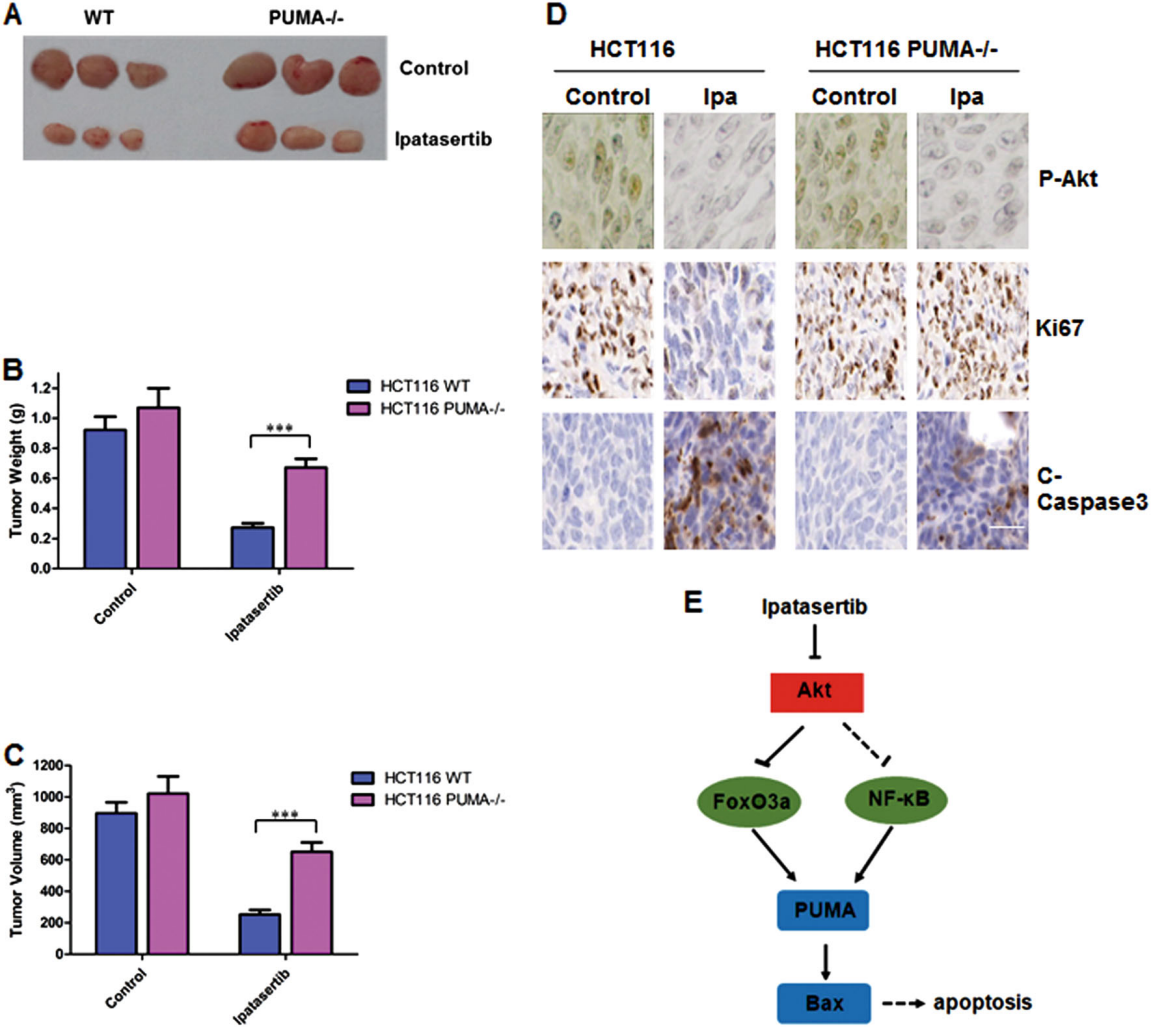
PUMA exhibited the antitumor effects of ipatasertib in vivo. Nude mice were injected s.c. with 1 × 10^6^ HCT116 WT or PUMA^−/−^. Once the tumor was measurable, mice were treated daily with 30 mg/kg ipatasertib by oral gavage, for 15 consecutive days. **a** Representative tumors at the end of the experiment, **b** tumor weight, and **c** tumor volume at indicated time points after treatment was calculated (*n* = 6 per group). Statistical significance is indicated for the comparison of ipatasertib-treated WT and PUMA^−/−^ tumor. Data represent the mean ± SEM of four independent experiments. ***P < 0.001 vs. WT. **d** IHC staining analyzed of P-Akt (S473), Ki67, and active Caspase-3 from paraffin-embedded sections of control or treated tumor tissues. Scale bar = 200 μm. **e** Molecular mechanism of ipatasertib induced apoptosis

Until now, we have proved that PUMA is indispensable in apoptosis induced by ipatasertib in vitro. It is important to study whether PUMA-mediated apoptosis is necessary for antitumor activity of ipatasertib in xenograft models. We generated subcutaneous tumors using WT and PUMA^−/−^ cells in a xenograft mice model. As a result, ipatasertib therapy significantly inhibited the growth of WT tumors, however, for the PUMA^−/−^ tumors, ipatasertib therapy caused some suppression, but not so markedly compared with that of WT tumors (Fig. 6a). Quantitative analysis of the weight and volume of tumors displayed similar trends (Fig. 6b, c). Immunohistochemistry staining shows that the expression of P-Akt reduced in both WT and PUMA^−/−^ tumors after ipatasertib therapy; Ki67 decreased obviously in WT tumors but had no big change in PUMA^−/−^ tumors; C-Caspase3 increased evidently in WT tumors, which had a little bit increase but not so sharp in PUMA^−/−^ tumors although Akt activation was inhibited (Fig. 6d). All these data suggested PUMA-dependent antitumor effects of ipatasertib in colon cancer.

## Discussion

Colon cancer is the third most common cancer and one of the most common cancer deaths globally, with roughly 1.2 million new cases and 600,000 deaths per year^39,40^. The efficiency of traditional chemotherapy for colon cancer treatment is rather limited due to the lack of specificity, such as 5-FU, oxaliplatin, and irinotecan^41^. Therefore, treatment therapeutic strategies need to be required. As we all know, Akt/PKB activation continuously in diverse cancers is very important for proliferation and survival of tumors. It has direct effects on the apoptosis pathway, for example, targeting the pro-apoptotic Bcl-2-related protein or regulating the transcriptional response to apoptotic stimuli via affecting on FoxO3a, NF-κB, and the activity of the p53 family^42–44^. Akt is a potent therapeutic target that may mediate resistance to the apoptotic effects of chemotherapy drugs therapy for a variety of cancers, including colon cancer^45,46^.

Ipatasertib is a highly selective, orally bioavailable Akt kinase inhibitor. Preclinical Pharmacology demonstrates ipatasertib exposure resulting in a dose-dependently pharmacodynamic effect and robust anti-tumor activity in a broad spectrum of human cancer cells in vitro and in vivo^47,48^. A variety of transplanted models with ipatasertib treatment showed effective antitumor activity, including PTEN-deficient prostate cancer and in metastatic breast cancer with PIK3CA H1047R mutation^49–52^. However, the antitumor effect and mechanism of ipatasertib in colon cancer remain to be illuminated.

In the present study, we found that the colon cancer growth was effectively suppressed after being treated by ipatasertib both in vitro and in vivo (Figs. 1, 4, 6 and Figure S1), showing the antitumor effect of ipatasertib in colon cancer therapy, which is consistent with the former researches in other human cancer cells^5,53^. Meanwhile, we investigated the mechanism on how ipatasertib inhibited proliferation of colon cancer cells. Comparing with other analysis, we confirmed ipatasertib induces PUMA-dependent but not p53-dependent apoptosis for the first time (Figs. 4, 6 and Figure S3).

Although it is well known that ipatasertib plays its anti-tumor activity through inhibiting Akt, how Akt activity status is affected by ipatasertib is still in controversial. Previous studies show that ipatasertib treatment enhanced the phosphorylation level of Akt in breast cancer (BT474) and some other cancer cells^38,54,55^, while recent report claims that ipatasertib stimulation decreased Akt phosphorylation in several different breast cancer cells even in the same cell line (BT474) as that in previous studies. Our results generated from colon cancer cells support the latter one, and we speculated that different cell types give us different phenomenon, and even for the same cell type, different results would also be obtained if the background of these cells is not exactly identical. However, the deep mechanism should be further studied and clarified.

Akt inhibition led to simultaneous activation of FoxO3a and p65, that up-regulated PUMA expression by directly binding to its promoter, subsequently initiated mitochondrial apoptosis (Figs. 2–4 and Figure S2). FoxO3a is the primary and p65 is the secondary regulator during ipatasertib-induced PUMA activation and apoptosis. In addition, PUMA/Bax axis is indispensable for ipatasertib only and combinational therapies in colon cancer (Figs. 4–6 and Figure S3). All these original data and indicated conclusions are novel points of the most significance.

For the first time, PUMA-dependent colon cancer growth suppression accompanied by the activationof FoxO3a and NF-κB by ipatasertib was observed (Figs. 1, 2). Another two Akt inhibitors afuresertib and perifosine also inhibited colon cancer growth and induced apoptosis (Figure S1D and S3C). For the mechanism, afuresertib plays its function partially through activating FoxO3a, p65, and PUMA, which is similar to ipatasertib. However, perifosine may play its role through some other ways, because it does not activate FoxO3a, p65, or PUMA at all (Figure S2C). Next, the interaction of PUMA promoter with FoxO3a or p65 was detected respectively by ChIP and we got a positive result (Fig. 3). FoxO3a and p65 did directly regulate PUMA on the transcriptional level and cause apoptosis that is dependent on PUMA/Bax axis (Figs. 3, 4 and Figure S3). These results above indicated FoxO3a/PUMA/Bax pathway accompanied by the p65/PUMA/Bax pathway to regulate ipatasertib-induced colon cancer apoptosis, which is distinct from previous reports involved the PI3K/Akt/mTOR pathway^38,56^. Notably, the influence of p65 on PUMA transcription is inferior to that of FoxO3a in HCT116 WT and p53^−/−^ cells, suggesting the FoxO3a/PUMA/Bax pathway plays a dominant role in ipatasertib-induced mitochondrial apoptosis. Besides, Bim may also play some role while Bcl-XL and Mcl-1 seems the primary anti-apoptotic factors. Autophagy or necroptosis was not found during this process.

As part of classical anti-tumor drugs show resistance during clinical application, most of the trials about cancer therapy are focused on a combination of ipatasertib and aclitaxel or hormonal recently. The latest clinical trials from tumor-patient samples show the combination of ipatasertib with paclitaxel as first-line therapy for triple-negative breast cancer^53^. The combined treatment of ipatasertib plus anti-microtubule chemotherapy, including vinorelbine, paclitaxel, eribulin, is contributed to anti-proliferative, pro-apoptotic, and anti-metastatic effect on human breast cancer cells^56^. In this study, we used the combination of ipatasertib with two conventional drugs, 5-FU and cisplatin, or with another novel TKI regorafenib. Because there is a common effect of that three drugs are up-regulating PUMA expression and promoting apoptosis in cancer cells^20,57,58^. As a result, the expression levels of PUMA and c-caspase3 increased obviously after combinational therapies, which shows a synergistic effect, compared with that of single treatment (Fig. 5). However, PUMA deficiency significantly abolished apoptosis induced by combinational therapies, indicating that PUMA is indispensable in the chemosensitization. Finally, the critical role of PUMA is also proved in vivo (Fig. 6).

In conclusion, our results demonstrated that the therapeutic effect of ipatasertib mediated by the cell autonomous process of apoptosis induction, exhibiting direct anticancer activity via inhibition of Akt and its downstream pathways. Importantly, the anticancer effect of ipatasertib is regulated by PUMA, progressing from Akt inhibition, FoxO3a and NF-κB activation, PUMA up-regulation, leading to PUMA/Bax dependent endogenous apoptosis, during which FoxO3a is the primary and NF-κB is the secondary regulator. PUMA expression induced by ipatasertib can be considered as a biomarker for clinical trials, which contributes to the important significance in future development and application.

## Materials and methods

### Cell culture and treatment

The human colon cancer cell lines, HCT116, RKO, DLD1, HT29, and NCM460, were obtained from American Type Culture Collection (ATCC). Human colon cancer cell line with p53^-/-^ (HCT116 p53-KO), PUMA^-/-^ (HCT116 PUMA-KO), and Bax^-/-^ (HCT116 Bax-KO) were generously provided by Dr. Bert Vogelstein (Johns Hopkins University, Baltimore, MD, USA). NCM460 were cultured in DMEM and other cell lines were routinely cultured in McCoy’s 5A modified media, supplemented with 10% fetal bovine serum (FBS), penicillin (100 units/ml), and streptomycin (100 mg/ml) in 5% CO_2_ at 37 °C in a humidified incubator. All agents of ipatasertib, afresertib, 5-FU, regorafenib, and cisplatin diluted with DMSO and perifosine diluted with water were added in the medium directly before detection. In the aspect of transfection experiments, the polyjet (Signgen Laboratories) transfection reagent was used following the supplier’s instructions. The medium was replaced with fresh culture medium after 5 h. Cells were then examined at 24–48 h after transfection.

### Antibodies and reagents

Primary antibodies against p53, Phospho-Akt (S473), Total-Akt, PUMA, Phospho-FoxO3a (S253), Total-FoxO3a, Phospho-p65 (S536), Total-p65, Bax, Noxa, Bid, Bim, Bcl-2, Bcl-XL, Mcl-1, Cox IV, Cleaved-Caspase3, β-actin LC3, Total-MLKL, and Phospho-MLKL (Ser358) were purchased from Cell Signaling Technology. Lipofectamine™ Reagent was purchased from Invitrogen. HRP-conjugated anti-rabbit and/or anti-mouse secondary antibodies and ECL-plus kit were from GE Healthcare. Ipatasertib and 5-FU were purchased from APP Pharmaceuticals. afuresertib and perifosine were purchased from Selleck Chemicals(Houston, TX). Regofenib and cisplatin were purchased from Axon Medchem. Other chemicals were mainly from Sigma. CCK-8 kit was from 7 Sea Biotech (Shanghai, China). The plasmid of expressing PUMA was kindly supplied by Jian Yu, PhD^9^.

### Real-time reverse transcriptase (RT) PCR

Total RNA was extracted with Tri-Reagent (Molecular Research Center, Cincinnati, OH) according to the manufacturer’s protocol. The amount and purity of the RNA were determined by spectrophotometry, and 3 μg of RNA from the colon cancers after ipatasertib treatment were used in each RT reaction. The performance of real-time qPCR was performed by our previous report that we described on C1000 Thermal Cycler CFX96 Real-time PCR Detection System (Bio-Rad)^59^.

### Cell viability and apoptosis assays

Colon cancer cells were cultured in 96-well microplate at a density of 5 × 10^3^ cells/well for 24 h. Cell viability was assessed with CCK-8 at indicated time post-treatment according to the manufacturer’s instructions. To estimate the viability of the cells, the absorbance of 450 nm (OD450) was measured with a 96-well plate reader (DG5032, Hua Dong, Nanjing, China).

For analysis of apoptosis by Hoechst 33258 (Invitrogen), colon cells were cultured on the coverslip of a chamber, rinsed with PBS, and then added in 500 ml of McCoy’s 5A containing 5 μg Hoechst 33258, incubated at 37 °C with 5% CO_2_ for 15 min. Apoptosis was detected through microscopic visualization of condensed chromatin and micronucleation.

For colony formation assays, equal number of cells after different treatments were plated into 6-well plates. Colonies were visualized by crystal violet staining 14 days after plating.

### Western blotting

Protein samples were extracted with RIPA buffer (10 mM Tris–Cl (pH 8.0), 1 mM EDTA, 0.5 mM EGTA, 1% Triton X-100, 0.1% sodium deoxycholate, 0.1% SDS, 140 mM NaCl). Equivalent protein samples (30 μg protein extract was loaded on each lane) were subjected to SDS -PAGE on 10% gel. The proteins were then transferred onto PVDF membranes (Millipore) and blocked with 5% non-fat milk for 1 h at room temperature. The membranes, probed with the indicated primary antibodies, were incubated at 4 °C overnight. Primary antibody was detected by binding horseradish peroxidase (HRP)-conjugated anti-rabbit or anti-mouse secondary antibody with an ECL plus kit. Detection was performed using the Odyssey infrared imaging system (LI-COR, Lincoln, NE).

To detect Bax multimerization, purified mitochondrial fractions were cross-linked with dithiobis(succinimidyl propionate) (DSP) (1 mmol/L), followed by Western blotting analysis.

### Flow cytometry

Human colon cancer cell line with HCT116 WT, p53^−/−^ (HCT116 p53-KO), PUMA^−/−^ (HCT116 PUMA-KO), and Bax^−/−^ (HCT116 Bax-KO) were suspended in 1 × 10^5^ cells/ml, and 5 μl Annexin V and 5 μl propidium iodide staining solution were added to 100 μl of the cell suspension. Then 400 μl binding buffer was added to cell suspension again. After the cells were incubated at room temperature for 10 min in the dark, stained cells were assayed and quantified using a FACSort Flow Cytometer (Beckman Coulter, Brea, CA, USA). Cell debris was excluded from the analysis by an appropriate forward light scatter threshold setting. Compensation was used wherever necessary.

### Chromatin immunoprecipitation

ChIP assay was performed using the Chromatin Immunoprecipitation Assay kit (Millipore, MA, USA) in accordance with manufacturer’s instructions with minor modifications. All the solutions used come from this ChIP Assay kit unless otherwise stated.

Briefly, after ipatasertib treatment, HCT116 WT or p53^−/−^ were fixed with 1% formaldehyde and lysed in warm SDS lysis buffer. The genomic DNA was obtained and sheared to 200–1000 bp by sonication on ice. Samples were precleared with Protein A-Agarose/Salmon Sperm DNA (50% Slurry) for 1 h at 4 °C with agitation. Then anti-FoxO3a antibody or anti-p65 antibody was added and incubated overnight on a shaker at 4 °C. Normal rabbit IgG (Invitrogen) was used as a negative control. The protein agarose/salmon sperm DNA (50% slurry) bead was then added to precipitate the antibody/protein/DNA complexes. After washed with serial wash buffers, DNA–protein immunocomplexes were eluted from the beads by elution buffer (1% SDS, 0.1 M NaHCO_3_) for 30 min. Finally, the protein–DNA cross-links were reversed to release DNA by incubation with 0.2 M NaCl at 65 °C for 4 h.

The total DNA was finally recovered from the samples by phenol/chloroform extraction and ethanol precipitation. Semi-quantitative PCR was then performed as described above. the precipitates were analyzed by PCR using primers 5′-GTCGGTCTGTGTACGCATCG-3′ and 5′-CCCGCGTGACGCTACGGCCC-3′ to amplify a PUMA promoter fragment containing putative FoxO3a or NF-κB sites^60^.

### Xenograft mouse model and treatment

HCT116 WT and PUMA^−/−^ were harvested, and 1 × 10^6^ cells in 0.2 ml of medium were implanted subcutaneously into the back of athymic nude female mice. Female 5-week-old nude mice (Vital River, China) were housed in a sterile environment with microisolator cages and allowed access to water and chow ad libitum. Mice were treated daily with ipatasertib at 40 mg/kg by oral gavage for 21 days treatment after 7 days. Calipers were used to monitor the tumor growth, volume was calculated by the formula: 0.5 × length × width^2^. Mice were euthanized when tumors reached ~1.0 cm^3^ in size. Tumors were dissected and fixed in 10% formalin and embedded in paraffin.

### Autopsy and histopathology

Animals were autopsied when the tumor has reached to the maximal size and tissues were collected and examined. Control and experimental tissue samples were fastened in 10% neutral-buffered formalin 24 h, transferred into 70% ethanol, and stored at 4 °C after washed once with 1× PBS. The tissues of ethanol dehydration embedded in paraffin by Lecia according to standard protocols. Sections (5 μm) were prepared for immunohistochemistry as we have previously described in the paper. Briefly, 1% hydrogen peroxide was used to blocked antigen retrieval with citric acid (pH 6.0) endogenous peroxidase activity. Primary antibody (anti-P-Akt (Ser473), Ki67, and C-Caspase3 antibody, dilution 1:200) was applied and incubated with secondary antibodies conjugated to peroxidase-labeled dextran polymer. Sections not exposed to secondary antibody served as negative controls.

### Statistical analysis

Statistical analyses were performed using GraphPad Prism V software. All assays were repeated independently more than three times. Data are represented as mean ± SEM in the figures. *P* values were calculated using the Student’s paired *t*-test.

## Electronic supplementary material

Figure S1

Table S1

Table S2

Figure S2

Figure S3

Supplemental materials
