# Supplementary figures and images for: Ipatasertib, a novel Akt inhibitor, induces transcription factor FoxO3a and NF-κB directly regulates PUMA-dependent apoptosis
Source: Cell Death Dis. 2018 Sep 5;9(9):911. doi: 10.1038/s41419-018-0943-9 (PMC6125489; doi:10.1038/s41419-018-0943-9)

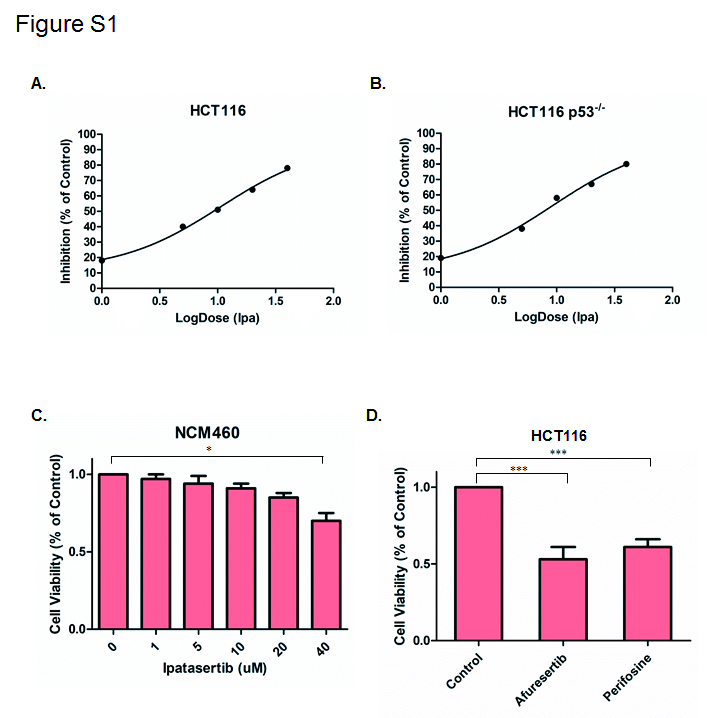

Supplement: Supplementary file 1 — Figure S1 [file 41419_2018_943_MOESM1_ESM.tif]

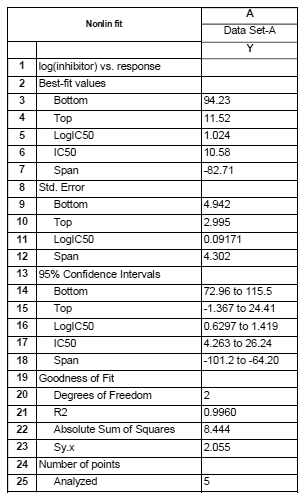

Supplement: Supplementary file 2 — Table S1 [file 41419_2018_943_MOESM2_ESM.tif]

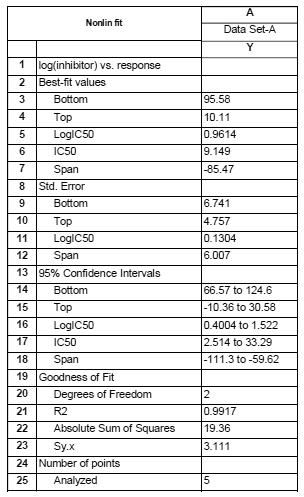

Supplement: Supplementary file 3 — Table S2 [file 41419_2018_943_MOESM3_ESM.tif]

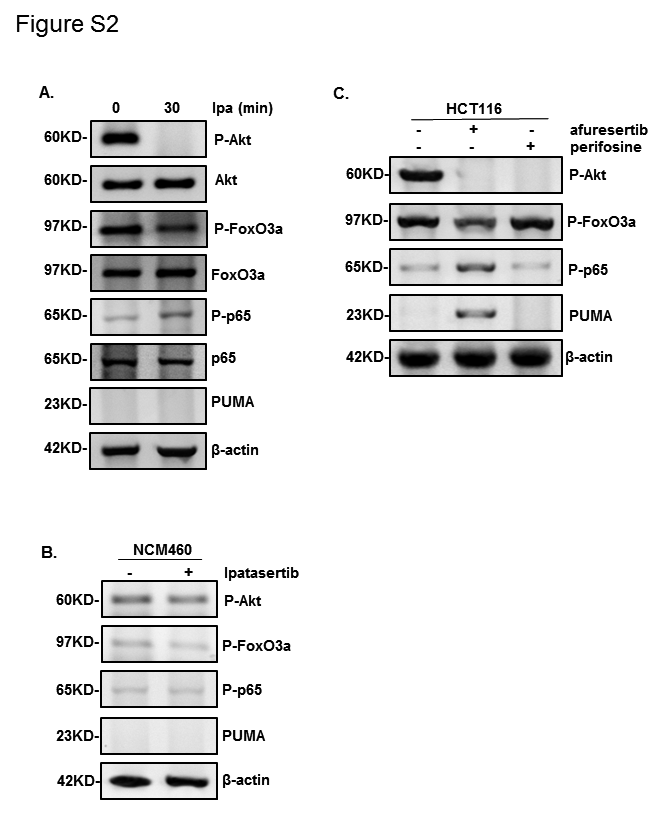

Supplement: Supplementary file 4 — Figure S2 [file 41419_2018_943_MOESM4_ESM.tif]

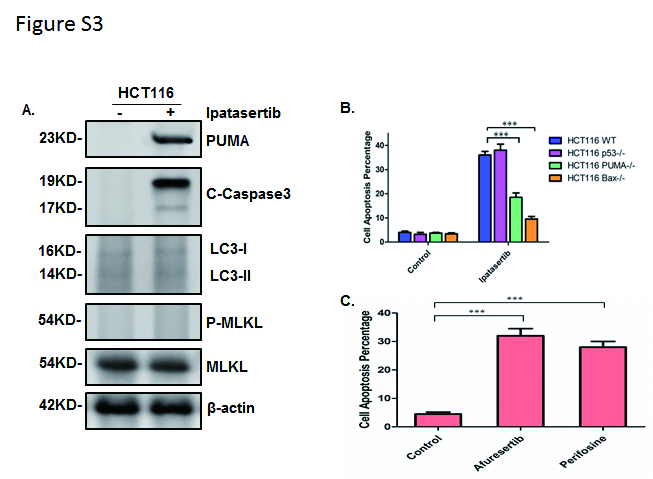

Supplement: Supplementary file 5 — Figure S3 [file 41419_2018_943_MOESM5_ESM.tif]
