# Supplementary material for: Ipatasertib, a novel Akt inhibitor, induces transcription factor FoxO3a and NF-κB directly regulates PUMA-dependent apoptosis
Source: Cell Death Dis. 2018 Sep 5;9(9):911. doi: 10.1038/s41419-018-0943-9 (PMC6125489; doi:10.1038/s41419-018-0943-9)
Supplement: Supplementary file 6 — Supplemental materials [file 41419_2018_943_MOESM6_ESM.docx]

**Figure S1**. **A and B.** IC50 values using CCK-8 assay were calculated in HCT116 WT (A) and in HCT116 p53^-/-^ (B). **C.** Cell viability of NCM460 were performed by CCK-8 assay after 10μM ipatasertib for 24h. **D.** Cell viability of HCT116 were detected the anti-proliferation of AKT inhibitors by CCK-8 assay after the treatment of afuresertib or perifosine alone for 24 hours.

**Figure S2.** **A.** AKT, FoxO3a, p65 and PUMA expression was analyzed by western blotting in HCT116 at 30 min after 10 μM ipatasertib treatment. **B.** The expression of P-AKT, P-FoxO3a, P-p65 and PUMA were detected by western blotting after the treatment of 10 μM ipatasertib for 24h in NCM460. **C****.** Western blotting analysis of P-AKT, P-FoxO3a, P-p65 and PUMA expression after the treatment of afuresertib or perifosine alone for 24h in HCT116.

**Figure S3.** **A.** The expressions of PUMA, C-Caspase3, LC3, P-MKLK (Ser 358), MKLK were analyzed by Western blotting after HCT116 were treated with ipatasertib for 24 h. **B.** Hoechst 33258 morphological examination of apoptosis in HCT116 WT, p53^-/-^, PUMA^-/-^ or Bax^-/-^. Cells were treated with 10 μM ipatasertib and incubated for 24 hours, then stained with Hoechst 33258. Relative apoptosis was calculated by counting condensed and fragmented nuclei normalized by untreated cells. Similar results were obtained from three independent experiments. Scale bar = 200 μm **C.** Apoptosis was analyzed by nuclear staining with Hoechst 33258 in HCT116 WT treated with ipatasertib, afuresertib or perifosine alone. Similar results were obtained from three independent experiments. Data represent the mean ± SEM of four independent experiments. ***P<0.001 vs. control.

**Table S1.** IC50 value of ipatasertib in HCT116.

**Table S2.** IC50 value of ipatasertib in HCT116 p53^-/-^.
